# Evaluation of changes of cardiac morphology and function in fetuses with ductus arteriosus constriction by Speckle-tracking echocardiography

**DOI:** 10.3389/fped.2023.1085352

**Published:** 2023-02-02

**Authors:** Tianjing Li, Jiancheng Han, Yanli Han, Xiaowei Liu, Xiaoyan Gu, Ye Zhang, Lin Sun, Ying Zhao, Shuang Gao, Xiuxiu Hao, Yihua He

**Affiliations:** ^1^ Department of Ultrasound, Beijing Anzhen Hospital, Capital Medical University, Beijing, China; ^2^ Department of Ultrasound, out-Patient Department, Communication University of China, Beijing, China

**Keywords:** fetal, echocardiography, premature ductus arteriosus constriction, speckle-tracking, fetal heart quantification

## Abstract

**Background:**

Premature ductus arteriosus constriction (DA Con) can result in right ventricular enlargement, right ventricular hypertrophy, and tricuspid regurgitation.

**Method:**

This study retrospectively analyzed 34 singleton fetuses that underwent fetal echocardiography with a diagnosis of DA Con (16 cases with mild to moderate, and 18 cases with moderate to severe) and 45 healthy fetuses. The morphology and function parameters of cardiac, as well as the 24-Segment of ventricles, were compared between the DA Con group and controls, and between the mild to moderate and moderate to severe groups, using the fetal heart quantification (FHQ) technology.

**Results:**

There were no significant difference in left ventricular parameters in DA Con group when compared to controls. Moreover, fetal 4CV-GSI was significantly reduced, as well as the sphericity index (SI), fractional shortening (FS), global longitudinal strain (GS) and fractional area change (FAC) of right ventricle, especially in the basal-middle segments. Compared with the mild to moderate group, LV-FS increased and RV-FS decreased in moderate to severe group.

**Conclusion:**

The results showed that the fetal heart in the DA Con group was different from the controls in morphology and function. FHQ technology provides a comprehensive assessment for the evaluation of cardiac morphological and functional changes in DA Con fetuses.

## Introduction

The ductus arteriosus (DA) connects the pulmonary with the systemic circulation and is integral to the maintenance of fetal circulation. Unlike other congenital heart malformations, which occur early during embryonic development, DA Con (ductus arteriosus constriction, DA Con) usually occurs in the second or third trimesters of pregnancy and is often associated with maternal use of prostaglandin synthase inhibitors; however, some cases are idiopathic. As a result, the fetal right heart circulation is compromised and can cause fetal right heart failure or even death in serious cases (1). It is reversible to some extent after the inducing factor is found and removed. Most of the fetuses we included in the current study were referred to our center because of increased right heart disproportion and tricuspid regurgitation found during routine prenatal echocardiography. We believe that the DA Con can also modify the shape and systolic function of the left and right ventricles (LV and RV, respectively) of the heart, in addition to causing disproportion of the right heart (2). The purpose of this study was to investigate te changes in end-diastolic global sphericity index (GSI) of the 4CV, as well as 24-segment spherical index (SI), fractional shortening (FS), global longitudinal strain (GS) and fractional area change (FAC) in fetuses with DA Con and to shed light on the effect of DA Con on fetal heart morphology and function, which could provide some guidance for the fetal assessment of segmental myocardial changes as well as the cardiac function.

## Materials and methods

### Study population

Fetuses with DA Con who were diagnosed by prenatal echocardiography at the Fetal Heart Disease Maternal Fetal Medical Consultation Center in our hospital from March 2013 to January 2020 were enrolled in this study. The Ethics Committee of Beijing Anzhen Hospital, Capital Medical University, approved this study. Written informed consent was obtained from the parents. Of the 34 fetuses enrolled, all singletons, 16 cases with mild to moderate and 18 cases with moderate to severe, without other congenital heart malformations that may cause stenosis or absence of ductus arteriosus. Forty-five singleton fetuses with a structurally normal heart of comparable gestational age (GA), and without concomitant abnormalities of the extracardiac system or growth restriction, during the same study period were randomly selected as controls. All the pregnant women have no underlying diseases.

### Diagnostic criteria

**FIGURE 1:**
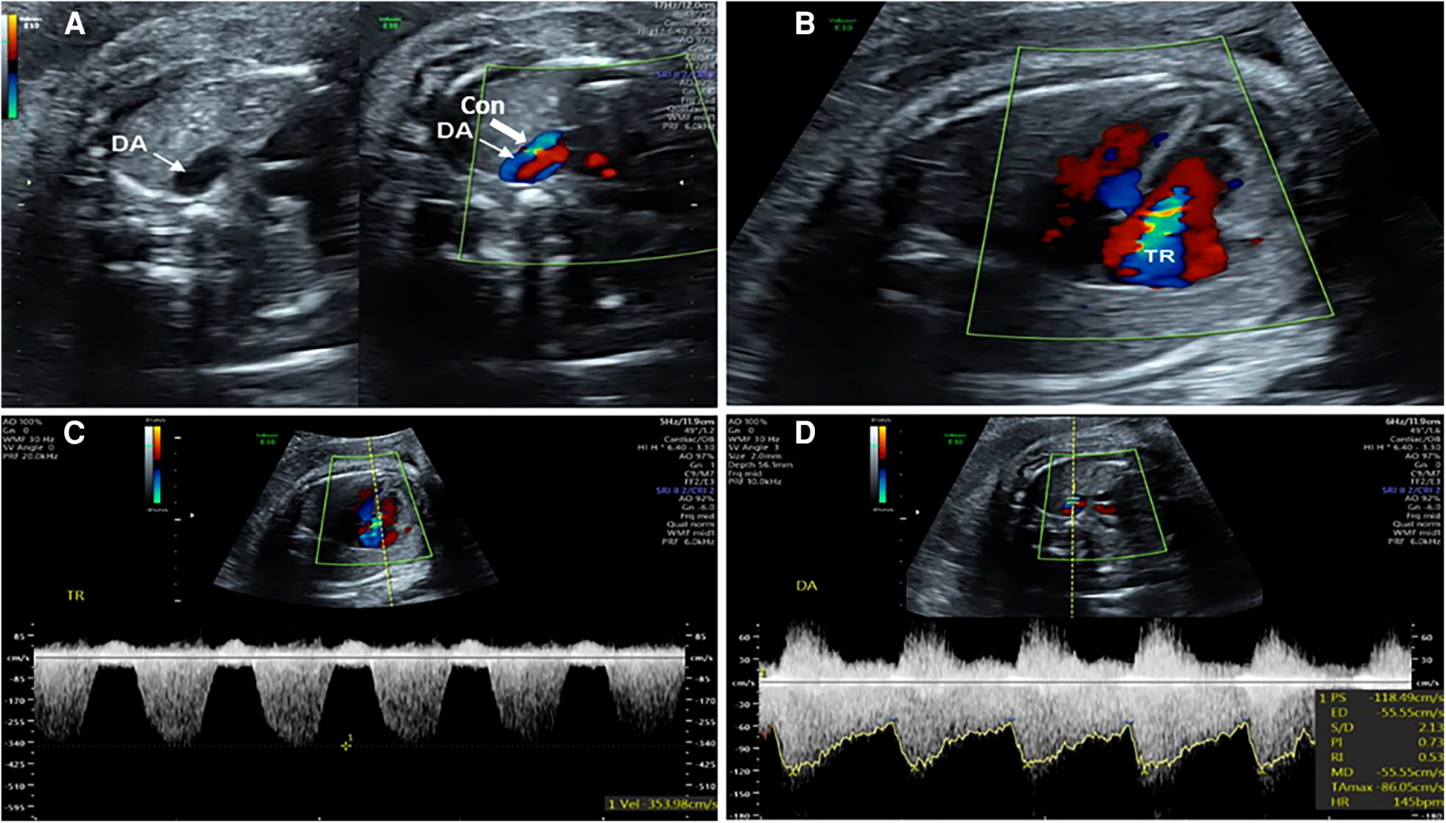
Echocardiograms of the fetuses with ductus arteriosus premature contraction (DA Con). (**A**) Narrowed ductus arteriosus; (**B**) Full-systolic tricuspid regurgitation (TR) on four-chamber view; (**C**) Velocity of TR; (**D**) Blood flow velocity and pulsatility index (PI) value of DA Con.

Two-dimensional echocardiography showing that the inner diameter of the DA was narrower than that of the aortic isthmus (Figure 1A). When compared to the LV, the RV was disproportionally increased in size and appeared to have decreased contractility; 2. Color Doppler demonstrated continuous turbulent, full-systolic tricuspid regurgitation (Figures 1B,C); 3. Pulsed Doppler showing peak systolic velocity of 200–300 cm/s (normal: 100–120 cm/s), diastolic peak velocity of 35 cm/s, and pulsatility index of the DA < 1.9 (normal: >2) (Figure 1D). Among them, 1.0 < pulsatility index (PI) ≤ 1.9 was defined as mild to moderate of DA Con, and PI ≤ 1.0 was defined as moderate to severe stenosis of DA Con.

### Exclusion criteria

(1) Presence of concomitant congenital heart malformation causing stenosis or absence of the DA; (2) Absence of standard image on 4CV.

### Fetal echocardiograms

All fetal echocardiographic examinations were performed using a Voluson E10 color Doppler ultrasound (GE Healthcare, Chicago, IL, USA) with a 4–8 mHz 2D/3D volume probe. Fetal biological parameters were measured during the 2nd or 3rd trimester, and the fetal heart was systematically examined under cardiac conditions. Segmental diagnosis was used to analyze the fetal cardiac structure. The final diagnosis of fetal echocardiography was made when a consensus was reached by two experienced physicians. Echocardiography was also performed following birth. The standard four chamber view (4CV) dynamic images were selected from the previous preserved images and imported in 4dv format, the frame rate was greater than 90 Hz. Then, after clicking the “Calculate” button, “Fetal HQ” is selected and the end-diastolic epicardial length and width of the 4-chamber view were measured. This was followed by activation of the speckle tracking contours of the end-systolic and end-diastolic images, identified from an M-mode generated from the 2D image, of 1 cardiac cycle. From the end-systolic and end-diastolic frames of the 2D image of the right and left ventricles, speckle tracking of the endocardial borders was used to compute the ventricular measurements of size, shape, and function (3, 4). When necessary, the ventricular contours were adjusted to adjust the endocardial borders at their origin at the base of the lateral wall and septum, along the ventricular and septal walls, as well as accurate identification of the apex of each chamber.

### Analysis method and index

1. The cardiac GSI was calculated by dividing the 4CV length by the 4CV width to evaluate the shape of the heart at end diastole. 2. The 24-segment SI was calculated by dividing the ventricular ED length by the 24-segment ED to evaluate the shape of the LV and RV. 3. Evaluation of RV and LV systolic function. 3.1. FAC. The formula is as follows: (end diastolic area—end collecting area)/end diastolic area * 100%. 3.2. GS. The calculation formula is as follows: endocardial length—endocardial length at diastole)/endocardial length at diastole * 100%. 3.3. The 24-segment FS. The formula is as follows: (end diastole width—endsystole width)/end diastole width * 100%.

### Statistical analysis

Measured data are expressed as the mean ± standard deviation (SD). SPSS 20.0 (IBM Corp., Armonk, NY, United States) was used for the statistical analysis. The differences in SI, FS, FAC, GS and 4CV GSI between the controls and DA Con group, and between the mild to moderate and moderate to severe groups, were compared by independent-sample *t*-tests.

## Results

### Participant characteristics

The clinical characteristics of the mothers and fetuses in the DA Con group and control group are summarized in Tables 1, 2. Pregnant women have no underlying diseases, and there were no significant difference in the maternal age, maternal BMI and GA between DA Con group and control group, as well as the mild to moderate and moderate to severe group (*P* > .05).

**TABLE 1:** Comparison of the clinical characteristics of the mothers and fetuses between the DA Con group and control group.

Group	N	Maternal age $\left(\bar{x}\pm s\right)$	Maternal BMI $\left(\bar{x}\pm s\right)$	Pregnant women with underlying diseases (N)	GA at first visit $\left(\bar{x}\pm s\right)$	Inner diameter of DA/PI $\left(\bar{x}\pm s\right)$	Peak velocity systolic/iastolic $\left(\bar{x}\pm s\right)$	Pregnancy outcome (N)		Delivery week N/ $\left(\bar{x}\pm s\right)$	
								Terminated pregnancy	Birth	Premature birth N/Weeks	Full term born N/Weeks
Control	45	31.5 ± 2.6	26.23 ± 1.70	0	32.76 ± 2.68	2.99 ± 0.29/2.53 ± 0.19	105.92 ± 7.17/21.6 ± 2.69	0	45	0/—	45/39.03 ± 1.58
DA Con	34	30.1 ± 3.7	26.81 ± 1.86	0	34.35 ± 2.97	1.46 ± 0.42/1.10 ± 0.42	197.56 ± 74.17/82.22 ± 58.93	0	34	4/33.66 ± 1.65	30/38.25 ± 1.44

**TABLE 2:** Comparison of the clinical characteristics of the mothers and fetuses between the mild to moderate and moderate to severe DA Con groups.

Group	N	Maternal age $\left(\bar{x}\pm s\right)$	Maternal BMI $\left(\bar{x}\pm s\right)$	Pregnant women with underlying diseases (N)	GA at first visit $\left(\bar{x}\pm s\right)$	Inner diameter of DA/PI $\left(\bar{x}\pm s\right)$	Peak velocity systolic/diastolic $\left(\bar{x}\pm s\right)$	Pregnancy outcome (N)		Delivery week N/ $\left(\bar{x}\pm s\right)$	
								Terminated pregnancy	Birth	Premature birth N/Weeks	Full term born N/Weeks
Mild to moderate	16	29.7 ± 3.1	26.67 ± 1.62	0	34.06 ± 2.56	1.60 ± 0.47/1.45 ± 0.26	198.77 ± 69.19/67.13 ± 55.94	0	16	1/34.29	15/38.13 ± 1.52
Moderate to severe	18	30.4 ± 2.7	26.92 ± 1.56	0	34.65 ± 2.45	1.35 ± 0.35/0.72 ± 0.15	196.25 ± 82.32/98.39 ± 59.72	0	18	3/33.45 ± 1.51	15/38.37 ± 1.41

### Comparision of the fetal heart shape between different groups

**FIGURE 2:**
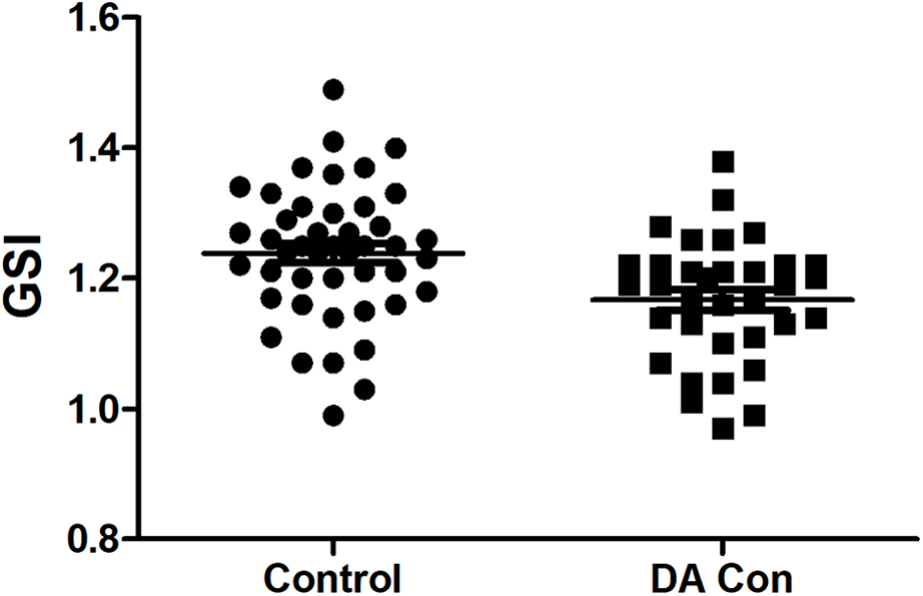
Scatter plot of global sphericity index (GSI) in the fetuses with ductus arteriosus premature contraction (DA Con) and control group (*P* < 0.05).

**FIGURE 3:**
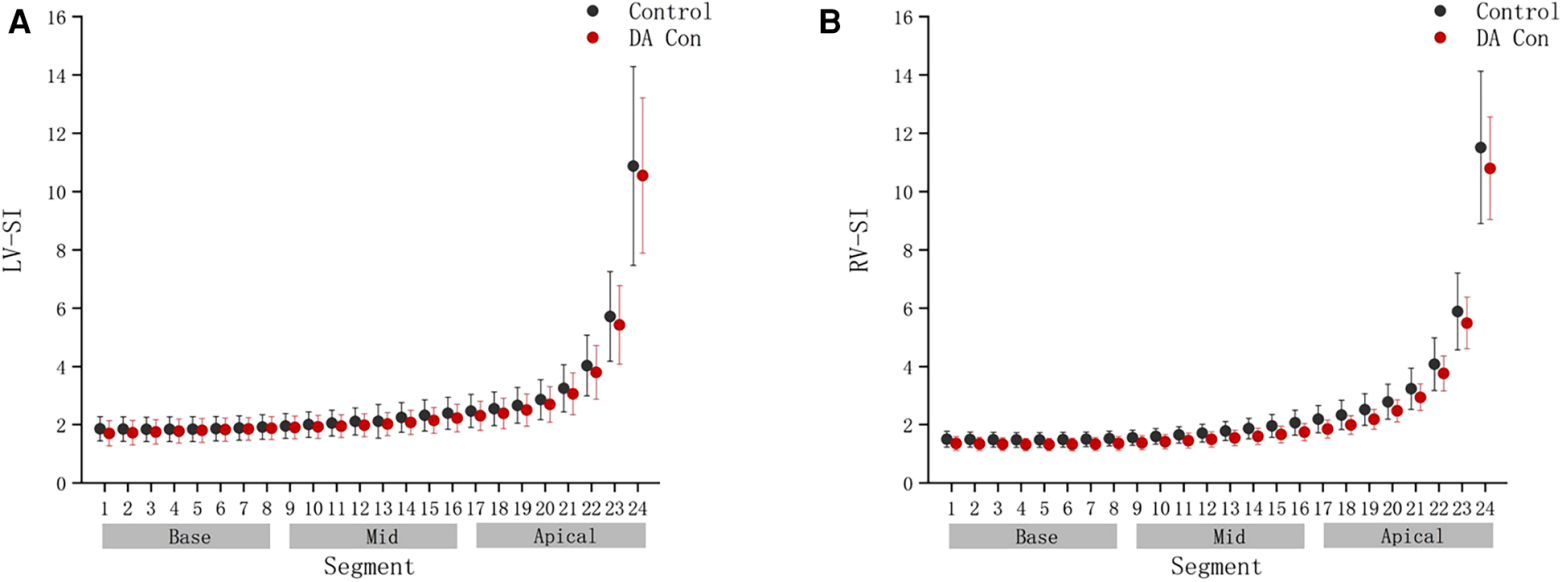
Comparison of ventricular 24-segment sphericity index (SI) from the base to the apex between the control group and ductus arteriosus premature contraction (DA Con) group. (**A**) Left ventricle (LV) 24-segment SI values in the DA Con group and control group (*P* < 0.05). (**B**) Right ventricle (RV) 24-segment SI values in the DA Con group and control group (*P* < 0.05).

Compared with the control group, the fetal 4CV GSI in the DA Con group was smaller than that in the control group(Figure 2) (*P *< .05), the fetal LV-SI and RV-SI was lower in 24 segments (Figures 3A,B), and the difference of RV-SI at segments 1–21 was statistically significant (*P* < .05).

**FIGURE 4:**
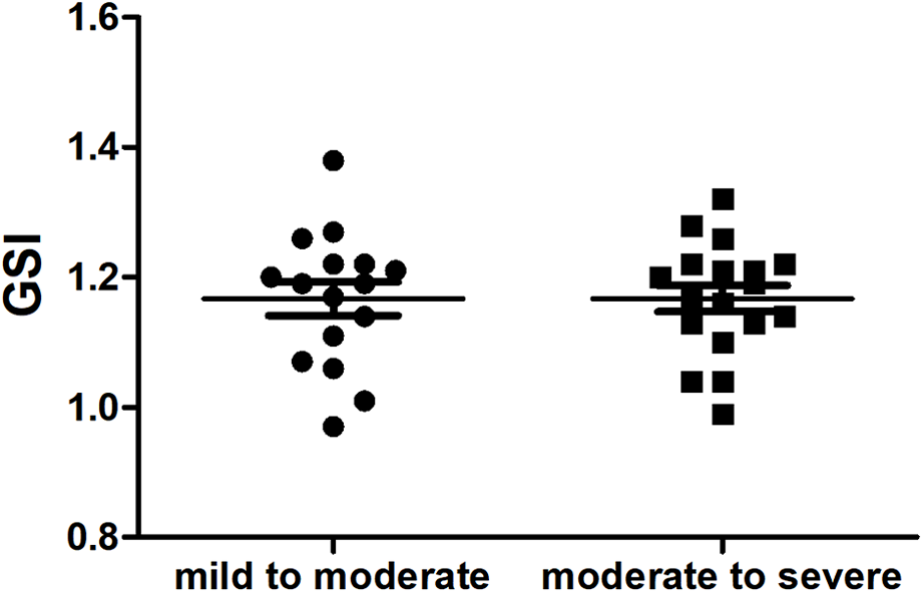
Scatter plot of global sphericity index (GSI) in the fetuses with mild to moderate and moderate to severe DA Con (*P* > 0.05).

**FIGURE 5:**
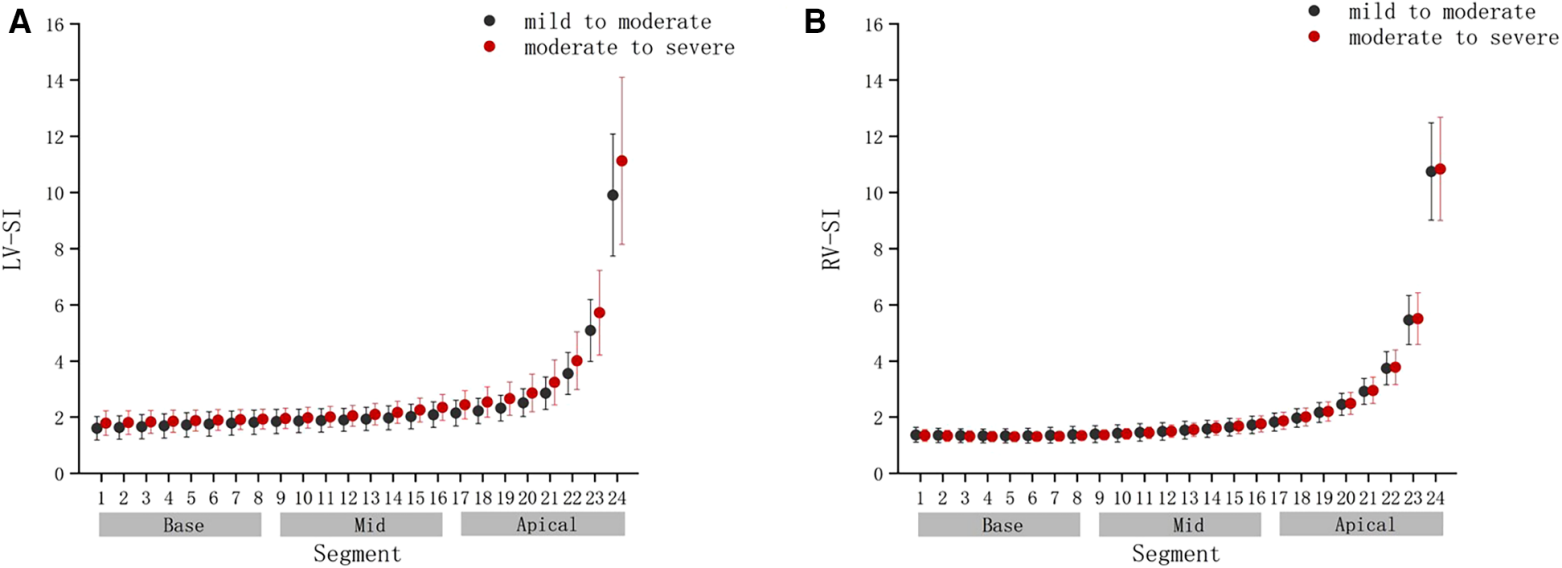
Comparison of ventricular 24-segment sphericity index (SI) from the base to the apex between the mild to moderate group and moderate to severe group. (**A**) Left ventricle (LV) 24-segment SI values in the mild to moderate group and moderate to severe group. (**B**) Right ventricle (RV) 24-segment SI values in the mild to moderate group and moderate to severe group.

Compared with the mild to moderate group, there were no significant change in the fetal 4CV GSI of the moderate to severe group (Figure 4) (*P* > .05), the same as the fetal LV-SI and RV-SI in 24 segments (Figures 5A,B) (*P* > .05).

### Comparision of the fetal heart function between different groups

**FIGURE 6:**
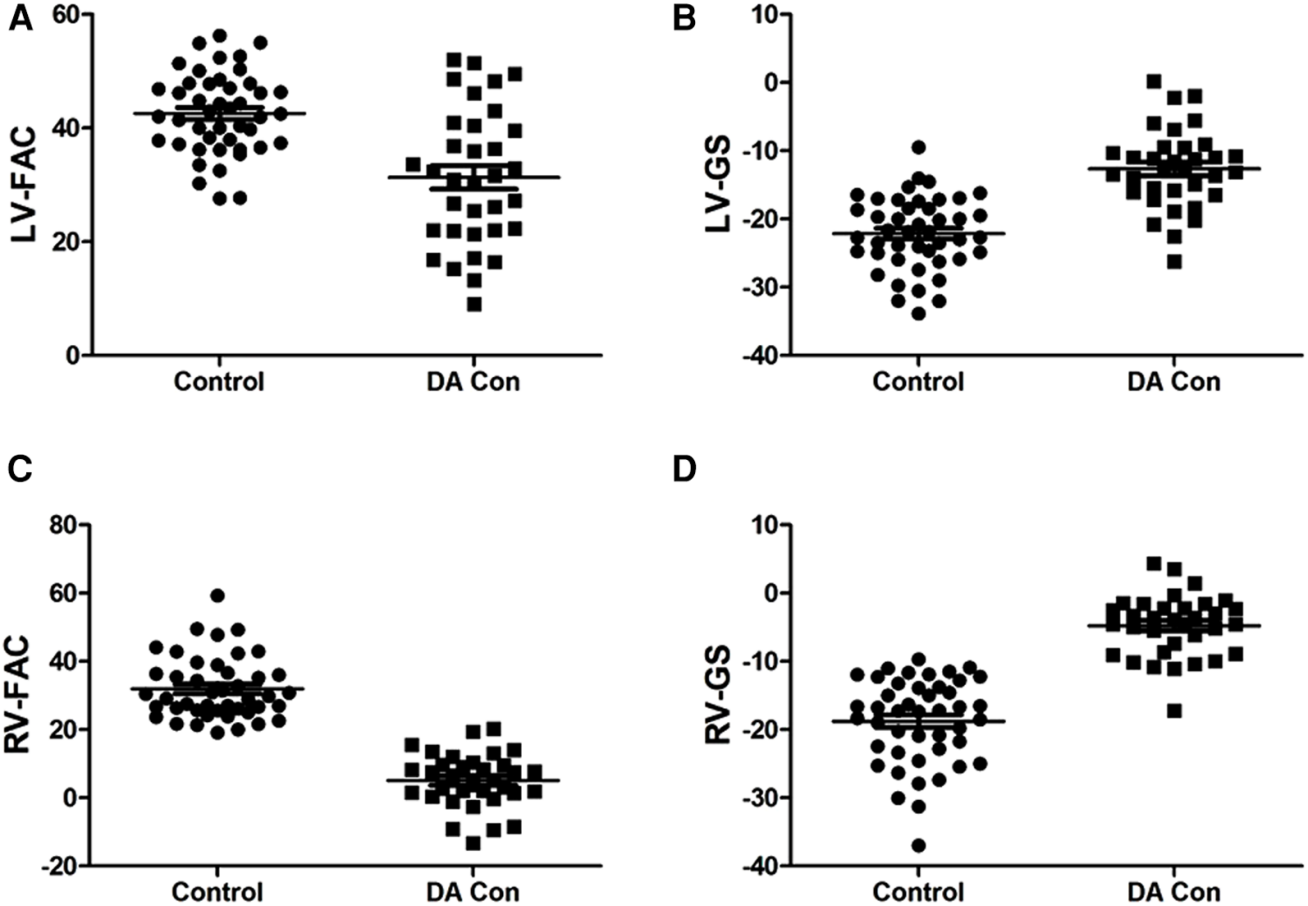
Scatter plot of left ventricle (LV)-fractional area change (FAC), LV-global strain (GS), right ventricle (RV)-FAC and RV-GS in the fetuses with mild to moderate and moderate to severe ductus arteriosus constriction (DA Con). (**A**) LV-FAC values in the control group and DA Con group. (**B**) LV-FS values in the control group and DA Con group. (**C**) RV-FAC values in the control group and DA Con group. (**D**) RV-GS values in the control group and DA Con group.

**FIGURE 7:**
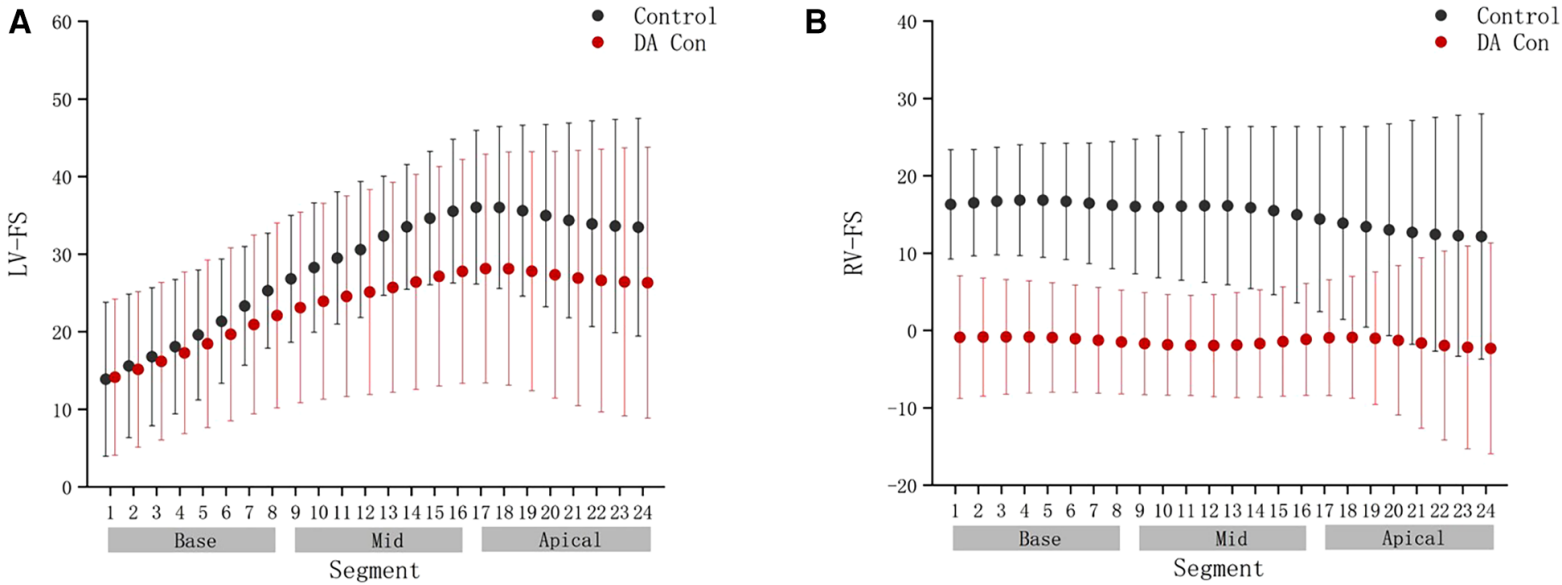
Comparison of ventricular 24-segment fractional shortening (FS) from the base to the apex between the control group and ductus arteriosus constriction (DA Con) group. (**A**) Left ventricle (LV) 24-segment FS values in the control group and DA Con group. (**B**) Right ventricle (RV) 24-segment FS values in the control group and DA Con group.

Compared with the control group, the fetal LV-FAC, LV-GS, RV-FAC and RV-GS were significantly decreased in the DA Con group(*P* < .05) (Figures 6A–D), and the fetal LV-FS and RV-FS were decreased in all 24 segments (Figures 7A,B), and the difference of LV-FS at segments 11–24, RV-FS at segments 1–24 were statistically significant (*P* < .05).

**FIGURE 8:**
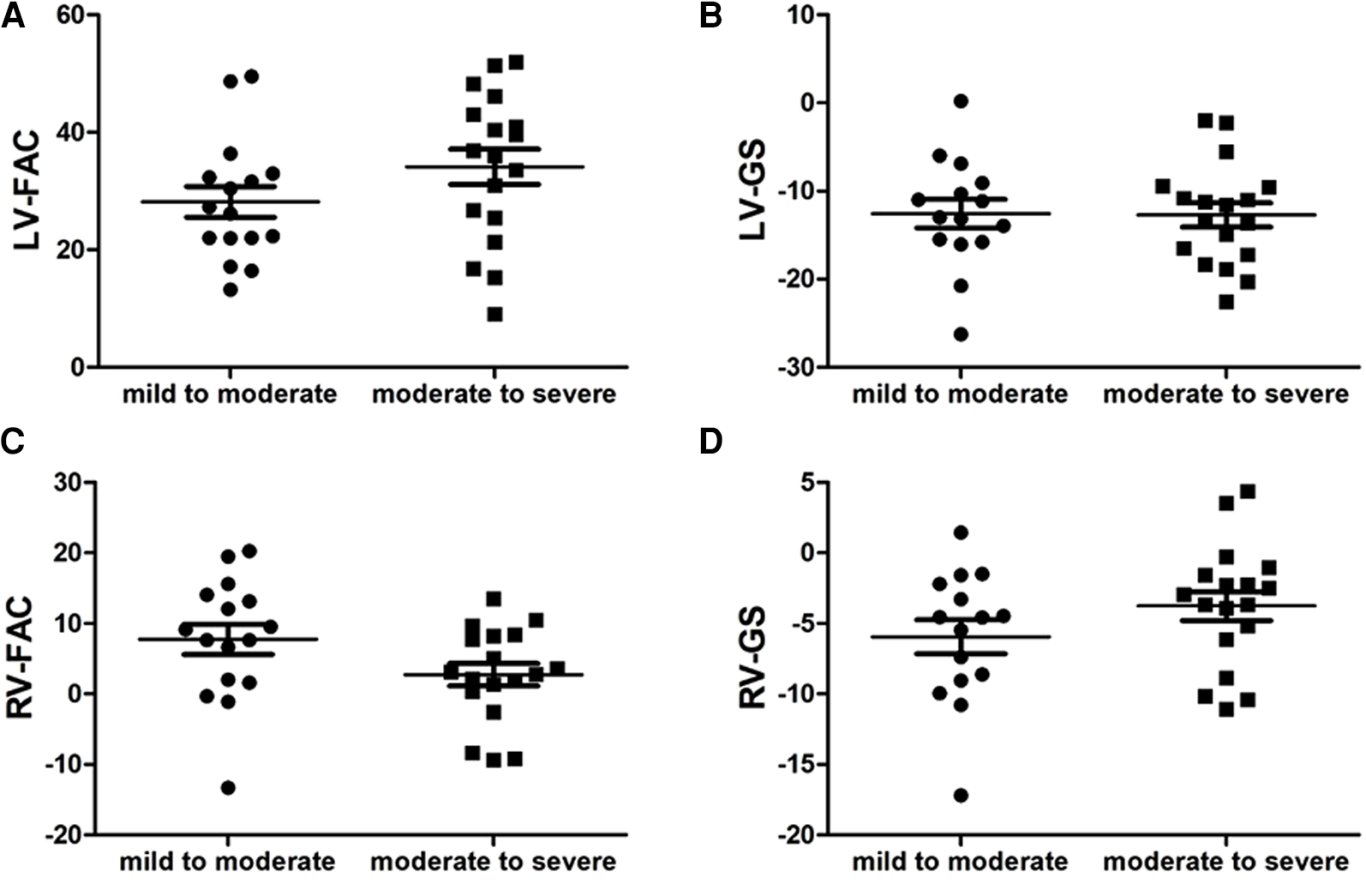
Scatter plot of left ventricle (LV)-fractional area change (FAC), LV-global longitudinal strain (GS), right ventricle (RV)-FAC and RV-GS in the fetuses with mild to moderate and moderate to severe ductus arteriosus constriction (DA Con). (**A**) LV-FAC values in the mild to moderate group and moderate to severe group. (**B**) LV-GS values in the mild to moderate group and moderate to severe group. (**C**) RV-FAC values in the mild to moderate group and moderate to severe group. (**D**) RV-GS values in the mild to moderate group and moderate to severe group.

**FIGURE 9:**
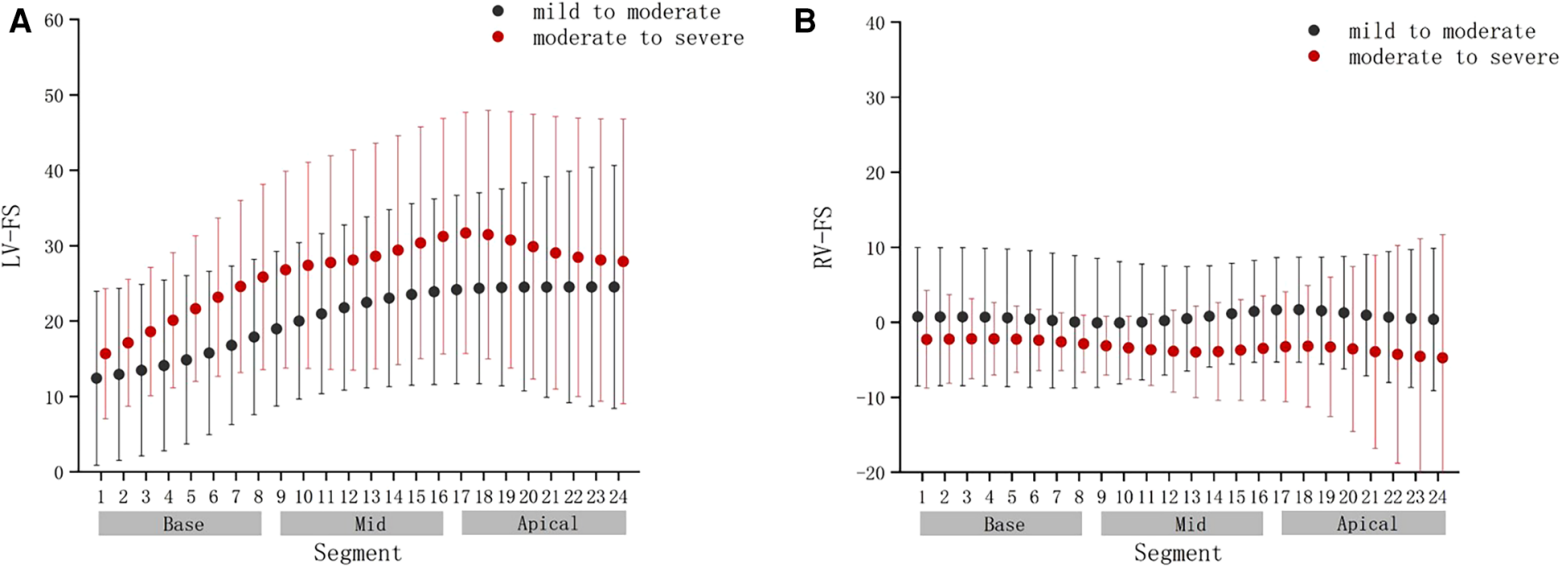
Comparison of ventricular 24-segment fractional shortening (FS) from the base to the apex between the mild to moderate group and moderate to severe group. (**A**) Left ventricle (LV) 24-segment FS values in the mild to moderate group and moderate to severe group. (**B**) Right ventricle (RV) 24-segment FS values in the mild to moderate group and moderate to severe group.

Compared with the mild to moderate group, there were no significant change in the fetal LV-FAC, LV-GS, RV-FAC and RV-GS of the moderate to severe group (Figures 8A–D) (*P* > .05), and the fetal LV-FS and RV-FS were increased in all 24 segments (Figures 9A,B), and the difference of LV-FS at segments 7, RV-FS at segments 14–17 were statistically significant (*P* < .05).

## Discussion

Fetal DA is anatomically and functionally completely different from that after birth. More than 80% of the blood from the right ventricle of the heart is drained directly into the descending aorta by the DA. Changes occur in the DA during the second or third trimester of pregnancy. The muscle thickens in the middle section of DA and the duct elongates, angles, and becomes tortuous. After birth, the DA closes and eventually becomes the ligamentum arteriosum. As pregnancy progresses, the sensitivity of the DA to factors prompting its contraction gradually increases, so DA Con usually appears in the second or third trimester of pregnancy (5). Studies have shown that premature constriction of DA is associated with decreased prostaglandin (PG), maternal nonsteroidal anti-inflammatory drug (NSAID) use, and consumption of grapes, green tea, coffee, and other dietary substances (6). The condition can be partially reversible when detected in a timely fashion (7). There are some rare idiopathic fetal DA Con cases without obvious causes. The incidence is very low, but the prognosis is worse than that of the fetal PDA related to NSAID (8). Prognosis may be related to the degree of DA constriction, the size of the foramen ovale, and the gestational age when the DA closes. The earlier the occurrence of DA stenosis, more severe stenosis, and limited opening of the foramen ovale affecting right-to-left shunt, the more serious the condition. Without timely intervention, it may lead to right heart failure, resulting in fetal death. Some scholars have found that if DA Con fetuses have hydrops, it is indicative of severe right heart dysfunction, as about 40% of them die imm ediately before or after birth, and therefore it is suggested that they should be delivered as soon as possible (9).

In the DA Con group, two-dimensional echocardiography showed narrowed DA and the dilated pulmonary artery on the 3-vessels view, RV dilation and decreased RV function on color Doppler, which were confirmed by changes in the pulse Doppler waveform, ductal flow velocity, and pulsatility index (10, 11). Previous studies on DA Con focused on the echocardiographic characteristics and diagnosis and did not discuss the difference of morphology and function between different segments of the myocardium. In the present study, fetal heart morphology and function were retrospectively assessed using the fetal heart quantification (FHQ) technology in the end-diastolic 4CV (12, 13), FS (14) and FAC (15), GS (16, 17) of the LV and RV in the DA Con group were lower than those in the control group, which indicated that the systolic functioning of LV and RV was lower than that in the control group. With the stenosis of DA aggravated, the compensation of systolic function of the LV was enhanced. This is because when fetal myocardium is damaged, not all segments of the whole ventricular wall appear at the same time. Even though the GS is reduced when the strain capacity of some segments of the ventricular wall is reduced, the myocardial strain of other segments may increase to compensate for this. The study data showed that the FS of RV 24-segment in the DA Con group was negative and the LV-FS increase at segment 11–24, which was considered to be indicative of RV dysfunction and LV compensatory contraction. Right ventricular wall moved passively as left ventricular systolic or diastole. Because the basal and apical segments were relatively fixed, the mid segment of the RV wall moved passively and in reverse with LV contraction and relaxation, with left-to-right shunt in systole and right-to-left shunt in diastole. Fetuses in the DA Con group had larger LV and RV and hearts as a whole, and smaller SI than those in the control group; the smaller the SI, the plumper and nearlyspherical the heart (18). This is due to the DA Con, as the RV blood cannot enter thedescending aorta through the DA. Because of the high pressure of pulmonary circulation, blood cannot pass through the pulmonary arteriole to the pulmonary venule, only to return to the RV, resulting in RV dilation, caused by the increase in the RV pressure load-originally from the RV shunted to the descending aorta through the DA part of the blood flow through the foramen ovale into the left heart system. Increased right-to-left shunt leads to increased LV volume load and enlarged cardiac chambers. Consequently, LV compensatory contraction and cardiac output increase. As the stenosis of the DA progressed, there were no significant change in the shape of fetal heart with moderate to severe DA Con and mild to moderate DA Con.

On the other hand, diastole is the active movement of myocardial energy consumption. We speculate that this may be due to increased acute RV afterload, leading to RV ischemia and fetal myocardial damage. The ventricular wall had almost no movement, and the diastolic function was reduced, resulting in restricted filling and little forward flow through the tricuspid valve. With the aggravation of DA stenosis, the right-to-left shunt of the fetus increased further. Heart rate did not increase significantly during the compensation period, and LV increased the blood output by enhancing contraction in the decompensation period. In addition, reduced LV systolic function, heart failure, decreased blood flow and increased pressure in the cardiac chambers lead to peripheral blood reflux, fetal pericardial effusion and tissue edema. Because the FHQ technology focuses on the analysis of systolic function, it needs to be combined with other methods to further study the changes of fetal diastolic function. The number of samples in this study was relatively small, so it is necessary to increase the sample size for further analysis and research, and to further confirm our hypothesis in combination with clinical practice.

DA Con occurs in the third trimester of pregnancy. The change in fetal heart load is an acute process and generally not combined with other organic lesions. Perinatal fetal prognosis is directly related to the choice of appropriate time of delivery after prenatal diagnosis. After birth, DA is physiologically closed, the shape and function of RV have changed greatly, and cardiac function can be restored over time. Therefore, the prognosis is generally good (19). At present, there is no consensus on the timing of termination of pregnancy for DA Con, mainly weighing the pros and cons of RV presure overload. Because increased right-to-left foramen ovale shunting can reduce the pressure load of the right cardiac system, some DA Con fetuses can be well compensated and do not develop right heart dysfunction. Therefore, it is not necessary to terminate the pregnancy immediately after diagnosis. Feeding difficulties and increased perinatal mortality may occur if the fetus is born too early and the various systems are underdeveloped. Late birth may lead to persistent pulmonary hypertension (20), which is not conducive to the recovery of the right heart function. Choosing the best time of delivery and reducing perinatal mortality is still a difficult problem that has not been completely solved.

The FHQ technique provides a new and comprehensive method to evaluate the size, shape, and function of the DA Con fetal heart. The measurement of these variables can provide important information for clinicians to assess myocardial changes sensitively, which can improve our understanding of DA Con fetal heart impairment. This can help clinicians to make prenatal clinical decisions and shape the clinical guidelines of the appropriate delivery timing in the future which can lead to improved fetal outcomes (21).

## Data Availability

The raw data supporting the conclusions of this article will be made available by the authors, without undue reservation.
